# Dynamic perfusion CT – A promising tool to diagnose pancreatic ductal adenocarcinoma

**DOI:** 10.1515/med-2021-0228

**Published:** 2021-02-05

**Authors:** Inga Zaborienė, Giedrius Barauskas, Antanas Gulbinas, Povilas Ignatavičius, Saulius Lukoševičius, Kristina Žvinienė

**Affiliations:** Department of Radiology, Lithuanian University of Health Sciences, Eiveniu str. 2, Kaunas, 50009, Lithuania; Department of Surgery, Lithuanian University of Health Sciences, Kaunas, Lithuania; Institute for Digestive Research, Lithuanian University of Health Sciences, Kaunas, Lithuania; Department of Visceral and Transplant Surgery, University Hospital of Zurich Zurich, Switzerland

**Keywords:** adenocarcinoma, computed tomography, deconvolution model, pancreas, perfusion

## Abstract

**Background and objective:**

This study deals with an important issue of setting the role and value of the dynamic computed tomography (CT) perfusion analysis in diagnosing pancreatic ductal adenocarcinoma (PDAC). The study aimed to assess the efficacy of perfusion CT in identifying PDAC, even isodense or hardly depicted in conventional multidetector computed tomography.

**Methods:**

A total of 56 patients with PDAC and 56 control group patients were evaluated in this study. A local perfusion assessment, involving the main perfusion parameters, was evaluated for all the patients. Sensitivity, specificity, positive, and negative predictive values for each perfusion CT parameter were defined using cutoff values calculated using receiver operating characteristic curve analysis. We accomplished logistic regression to identify the probability of PDAC.

**Results:**

Blood flow (BF) and blood volume (BV) values were significant independent diagnostic criteria for the presence of PDAC. If both values exceed the determined cutoff point, the estimated probability for the presence of PDAC was 97.69%.

**Conclusions:**

Basic CT perfusion parameters are valuable in providing the radiological diagnosis of PDAC. The estimated BF and BV parameters may serve as independent diagnostic criteria predicting the probability of PDAC.

## 1 Introduction

Pancreatic ductal adenocarcinoma (PDAC) is the most common primary malignancy of the pancreas, accounting for about 80% of malignant pancreatic tumors and is characterized by dismal prognosis. Contrast-enhanced multidetector computed tomography (MDCT) maintains the fundamental imaging modality for identification, characterization, and staging of PDAC [1].

Using MDCT as a diagnostic test, approximately 11% of ductal adenocarcinomas are missed, as the density of the pancreas parenchyma and the tumor may appear very similar. Differential diagnosis of paraduodenal pancreatitis, chronic focal pancreatitis, and pancreatic cancer is also complicated [2]. It has been reported that small or well-differentiated PDACs lack characteristic CT features; therefore, these lesions could be missed or misdiagnosed [3,4,5]. A reliable noninvasive method that allows improving accuracy in diagnosing PDAC is desirable. Perfusion CT could be feasible in improving the detection rate and could also be useful for better characterization of PDAC. Perfusion CT is based on contrast media kinetics in the tumor tissue supplying quantitative information on tissue hemodynamics. It has been reported to be a reliable imaging modality differentiating benign and malignant lesions, evaluating response to treatment, even describing angiogenesis [6,7,8,9,10,11].

Furthermore, this functional imaging could be useful in the evaluation of tumor aggressiveness, influencing treatment strategy, and patient management [12].

This study deals with an important issue of setting the role and value of the dynamic CT perfusion analysis in diagnosing pancreatic adenocarcinoma. Early diagnosis of pancreatic cancer is directly associated with increased resectability with curative intent. However, in the resectable solid pancreas tumor, the biopsy is not recommended; consequently, there is no possibility to unequivocally establish the diagnosis of cancer preoperatively. Noninvasive identification of tumors would be desirable in terms of improved patient selection.

The study aimed to estimate CT perfusion parameters in CT-defined tumorous foci and to evaluate if any of these parameters are of value in diagnosing PDAC.

We analyzed the role of perfusion CT basic parameters in predicting PDAC in our study. Fifty-six patients with PDAC in the head of the pancreas were included and all of them underwent perfusion CT imaging. According to the literature, shallow breathing during perfusion imaging is acceptable [12]. All our patients were breathing during data acquisition. It has been shown earlier that there is no difference in calculating perfusion parameters while placing one big region of interest (ROI) or several smaller ones on the tumor [12]. We placed four ROI on different parts of the tumor, ensuring the tumor ROI remained on the tumor tissue, excluding cystic areas or the main pancreatic duct, and calculated the average values of the four basic perfusion parameters.

## 2 Methods

This was a prospective clinical study, in which 242 subjects with suspected PDAC were evaluated from June 2015 to September 2018. All the patients underwent CT imaging with contrast enhancement as a standard protocol for confirmation/rejection of the diagnosis.

The diagnostic criteria for PDAC were as follows: ill-defined hypoattenuating mass when compared with the surrounding parenchymal tissue, abrupt termination of the main pancreatic duct (MPD) with upstream dilatation, double-duct sign, mass effect, and decreased fat displacement as compared with the remaining pancreatic parenchyma [13].

**Figure 1:**
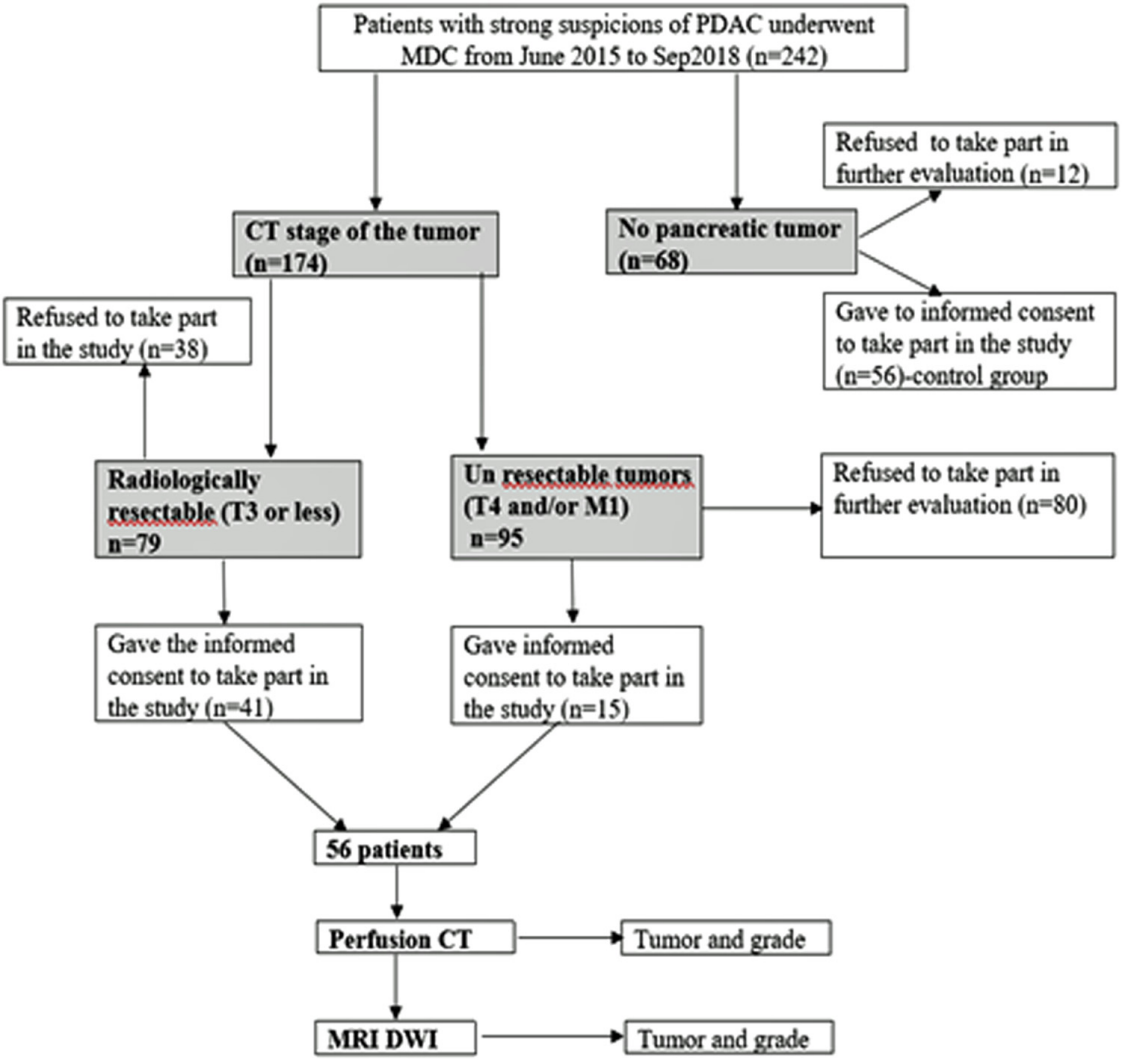
Flowchart of the patient selection process.

The pancreatic tumor was diagnosed in 174 patients following the MDCT examination. Less than one-third (56) of these patients signed informed consent for further evaluation with perfusion CT. In 68 patients, MDCT revealed no pancreatic tumor. However, 56 of them signed informed consent for further evaluation with perfusion CT and maintained as a control group. The flowchart of the patient selection process is shown in Figure 1.

The exclusion criteria for perfusion CT were as follows: patients with impaired renal function, cystic or hypervascular pancreatic lesions, any type of pancreatitis, pancreatic lesion smaller than 1 cm in size (because of the disability to put four independent ROIs on the lesion), and patients who refused to sign informed consent.

The Regional Biomedical Research Ethics Committee approved the study and all the patients gave their informed consent (study protocol No. BE-2-22, as of 13 May 2015). The patients were managed at the Department of Surgery and the Department of Gastroenterology from June 2015 to September 2018. All the study group patients had their PDAC diagnosis verified, by either histopathological examination of surgical specimen or biopsy of the tumor.

### 2.1 Computed tomography imaging

All the subjects underwent an MDCT scan using the GE light Speed Pro 64 CT (GE Healthcare, Milwaukee, Wis) scanner and perfusion CT (p_CT) of the tumor and nontumorous pancreatic parenchyma. MDCT scanning was performed with a power injector (Ulrich Missouri, Ulrich GmbH & Co. KG, Ulm, Germany), 80–100 mL of nonionic intravenous contrast media (Ultravist 370; Bracco, Milan, Italy), at a rate of 4.5 mL/s.

The hypodense or isodense mass or areas with mass suspicion were considered as lesions on MDCT and were analyzed further with perfusion CT. If no mass was visible, ROI was placed at the site of the MPD abruption.

The subject was immobilized for 15 min on the table to cleanse pancreatic parenchyma of the contrast media, given during MDCT, and to give instructions of slow breathing during p_CT.

All the subjects had a local perfusion assessment (based on the deconvolution model of perfusion) involving an evaluation of blood flow (BF) (mL min^−1^ 100 g^−1^), blood volume (BV) (mL 100 g^−1^ of tissue), mean transit time (MTT) (s), and permeability surface (PS) (mL min^−1 ^100 g^−1^).

Pancreatic perfusion was performed within a period of 50 s after a bolus injection of 50 mL of nonionic intravenous contrast media (Ultravist 370; Bracco, Milan, Italy) at a rate of 5 mL/s, applying 120 kVp, 150 mA s, 5 mm slice thickness, and 300 mm FOV. The total number of p_CT images was 792. The lowest possible value of *z*-axis coverage was selected to minimize the radiation dose.

Raw data images were evaluated at a CT perfusion software-equipped workstation, using the deconvolution model (AW Workstation, GE Healthcare). The arterial input function was determined by placing a circular ROI at the abdominal aorta. The area of the ROI was standardized at 50 mm^2^. Four round ROIs (50 mm^2^) were placed on the different parts of the tumor and on nontumorous pancreatic tissue for perfusion measurements. The mean value of these four ROIs was calculated for both tumor and nontumorous/fibrotic parenchyma. Image analysis was performed by two independent radiologists with 7 and 17 years of experience in abdominal radiology. The calculations were compared and, observing no major discrepancies in the obtained results, the mean values of both calculations were used for further analysis.

Histopathological analysis of surgery specimens or biopsy material was performed on a routine basis at the Department of Pathology, Medical Academy, Lithuanian University of Health Sciences. The pathologists were blinded to the perfusion CT results.

**Figure 2:**
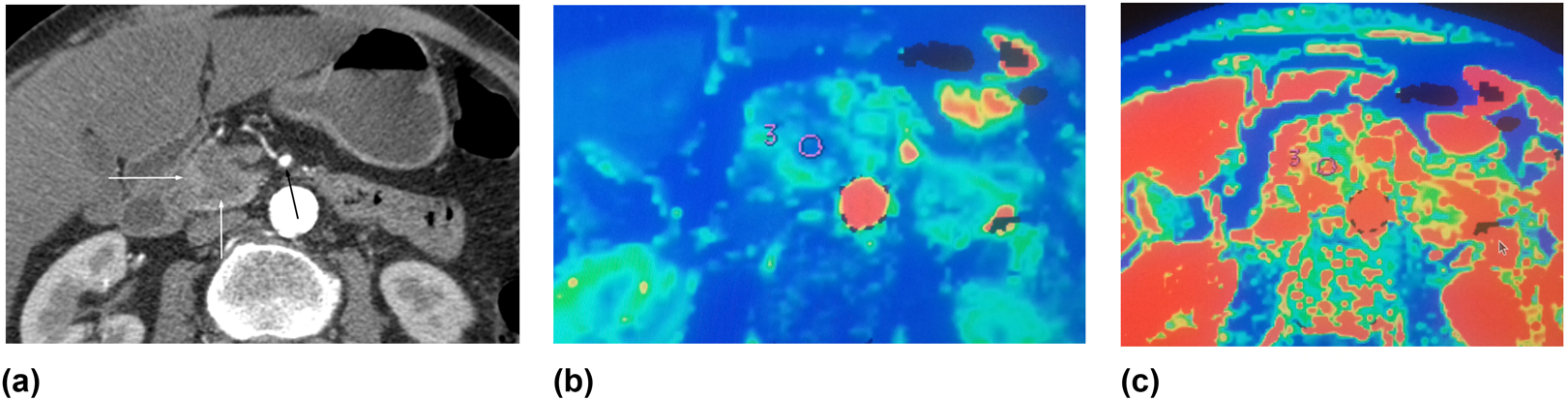
(a–c) Poorly differentiated (G3) PDAC in the head of the pancreas. Hypodense tumor in the head of the pancreas: (a) contrast-enhanced CT image, axial plane, arterial phase; hypodense mass in the head of the pancreas is seen (white arrows); no spread to the peripancreatic fatty tissue is detected; no contact with the superior mesenteric artery (SMA) (black arrow); a radiologically resectable tumor; (b and c) perfusion CT images of the same patient; third ROI of 4 is shown; (b) significantly reduced BF (29.24 mL min^−1^ 100 g^−1^) in the tumor was detected; (c) reduced blood volume of the tumor – 6.13 mL 100 g^−1^ (ROI). The examination of the surgical specimens revealed poorly differentiated PDAC (G3).

**Figure 3:**
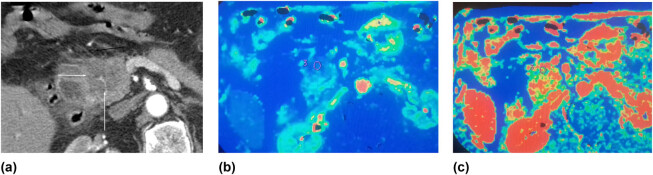
(a–c) Moderately differentiated (G2) PDAC in the head of the pancreas. Contrast-enhanced CT images of PDAC (G2) in the head of the pancreas: (a) arterial phase – a hypodense tumor in the head of the pancreas (white arrows) with infiltration of the peripancreatic fatty tissue (black arrow); (b–c) perfusion CT images of the same patient with G2 (moderate differentiated) tumor: (b) reduced BF in the tumor was detected (blood flow 43.24 mL min^−1^ 100 g^−1^); (c) reduced blood volume of the tumor – 7.6 mL 100 g^−1^ (ROI).

Figure 2a–c shows the data of the patient with PDAC in the head of the pancreas. At the time of imaging, the tumor was resectable and the patient underwent the Whipple procedure. Histopathological examination revealed poorly differentiated (G3) PDAC. Figure 3a–c shows the data of the patient with moderately differentiated (G2) PDAC in the head of the pancreas.

### 2.2 Statistical analysis

The Kolmogorov–Smirnov test and the Shapiro–Wilk test were used to determine whether the continuous variables were normally distributed. The data were expressed as mean values (SD – standard deviation) or median (in cases of abnormal distribution, for BF and PS; interquartile range Q1–Q3). Variables conforming to a normal distribution were compared via the Student’s *t*-test; otherwise, the Mann–Whitney U-test was used for comparison. Diagnostic accuracy was assessed as the area under the receiver operating characteristic (ROC) plot. Sensitivity, specificity, and positive and negative predictive values for each perfusion CT parameter in the prediction of the presence of PDAC were calculated using cutoff values chosen based on ROC curves. Discriminant function analysis was used to identify the differences between the control and study groups. At each step, the variable that minimizes the overall Wilks’ lambda was entered. We performed logistic regression to identify the probability of PDAC. All calculations were performed using SPSS for Windows 23.0 software and Microsoft Excel 16. The *P*-value less than or equal to 0.05 was considered as statistically significant.

## 3 Results

### 3.1 General group characteristics

The study group included 56 patients with PDAC in the head of the pancreas: 22 men (39.3%) and 34 women (60.7%). The mean age was 66.86 years (11.86). The mean size of the tumor was 34.98 (9.99) mm (range 13–58 mm). The control group consisted of 56 patients with nontumorous pancreatic tissue: 27 men (48.2%) and 29 women (51.8%). The mean age of the patients in this group was 64.86 years (12.26). There was no difference in age or gender between the groups (*P* = 0.382 and *P* = 0.341, respectively).

### 3.2 Perfusion CT parameters

The median BF and PS values (parameters with abnormal distribution) and the mean value (SD) of BV and MTT for the nontumorous pancreatic tissue were 138.129 (mL min^−1^ 100 g^−1^), 51.223 (mL min^−1 ^100 g^−1^), 16.139 (6.386) (mL 100 g^−1^), and 8.961 (4.550) (s), respectively.

The median values of BF and PS for PDAC patients were 41.740 (mL min^−1^ 100 g^−1^) and 24.097 (mL min^−1^ 100 g^−1^); the mean values (SD) of BV and MTT were 7.941 (4.315) (mL 100 g^−1^) and 12.473 (6.273) (s), respectively.

The median values of BF and PS and the mean value of BV were lower in PDAC compared with the nontumorous parenchymal tissue (*P* < 0.001), and the mean value of MTT was higher in PDAC, compared with the control group (*P* = 0.001). The distribution between perfusion parameters in nontumorous pancreatic parenchyma (control group) and PDAC is presented in Table 1.

**Table 1:** Perfusion CT parameters with SD in normal parenchymal tissue and PDAC

Parameters	Nontumorous tissue	PDAC	*P*-value
BF (mL min^−1^ 100 g^−1^); median (IQR)	138.13 (125.42–166.10)	41.74 (32.65–71.98)	<0.001
BV (mL 100 g^−1^); mean (SD)	16.14 (6.39)	7.94 (4.32)	<0.001
MTT (s); mean (SD)	8.96 (4.55)	12.47 (6.27)	0.001
PS (mL min^−1^ 100 g^−1^); median (IQR)	51.22 (28.17–79.36)	24.71 (16.00–42.79)	<0.001

Median in abnormal distribution of parameters.

Abbreviations: SD, standard deviation; BF, blood flow; BV, blood volume; MTT, mean transit time; PS, permeability surface; SD, standard deviation; PDAC, pancreatic ductal adenocarcinoma; IQR – interquartile range.

We used discriminant function analysis to identify the differences between PDAC and the nontumorous parenchymal tissue. We included four main perfusion variables (BF, BV, MTT, and PS) to establish the most important ones.

At each step, the variable that minimizes Wilks’ lambda was entered, detecting BF and BV as significant independent discriminators (Table 2). Fisher’s classification coefficient for classification function was as follows:

{ƒ}_{\text{tumor}}=-3.332+0.027\times \text{BF}+0.058\times \text{BV}.

**Table 2:** BF and BV values are significant discriminators between groups of patients with nontumorous parenchymal tissue and PDAC

Wilks’ lambda									
Exact F									
Step	Entered	Statistic	Df1	Df2	Df3	Statistic	Df1	Df2	Sig.
1	BF	0.336	1	1	110.000	217.431	1	110.000	*P* < 0.001
2	BV	0.314	2	1	110.000	118.885	2	109.000	*P* < 0.001

Abbreviations: BF, blood flow; BV, blood volume.

**Figure 4:**
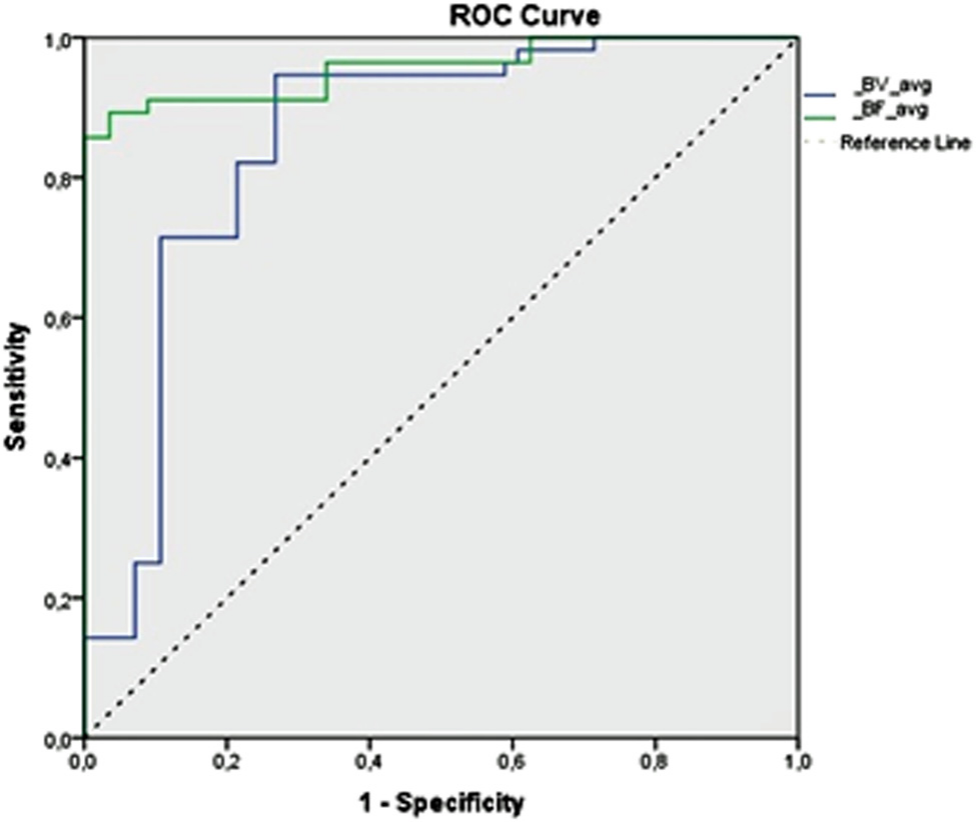
Receiver operating characteristic (ROC) curve analysis for blood flow (BF) and blood volume (BV) perfusion parameters. BF value less than 98.443 mL min^−1 ^100 g^−1^ (area under the curve [AUC] = 0.957) and BV value less than 14.319 mL 100 g^−1^ (AUC = 0.852) are shown to be good predictors for the presence of PDAC.

Particular cutoff values for BF, BV, MTT, and PS were calculated using ROC analysis to practically differentiate PDAC from the nontumorous pancreas tissue. Based on the ROC analysis, the area under the curve for BF and BV was outstanding and excellent, respectively (Figure 4).

The BF value less than 98.443 mL min^−1^ 100 g^−1^ was concomitant to the presence of PDAC with an 89.3% sensitivity and a 96.4% specificity (area under curve = 0.957, outstanding); the BV of less than 14.319 mL 100 g^−1^ was concomitant to the presence of PDAC with a 94.6% sensitivity and a 73.2% specificity (area under curve = 0.852, excellent). Cutoff points for all perfusion parameters with their predictive values are presented in Table 3.

**Table 3:** Analysis of sensitivity, specificity, PPV and NPV, and ROC for averaged BF, BV, MTT, and PS for the presence of PDAC

PDAC	BF	BV	MTT	PS
AUC	0.957	0.852	0.653	0.749
Cutoff point	<98.443	<14.319	>15.248	<43.506
Sensitivity	89.3%	94.6%	48.2%	80.4%
Specificity	96.4%	73.2%	89.3%	62.5%
PPV	96.2%	78%	82%	68.2%
NPV	90%	93%	63%	76.1%

Abbreviations: PPV, positive predictive value; NPV, negative predictive value; ROC, receiver operating characteristic; BF, blood flow; BV, blood volume; MTT, mean transit time; PS, permeability surface; AUC, area under the curve.

### 3.3 Independent diagnostic criteria of PDAC

**Figure 5:**
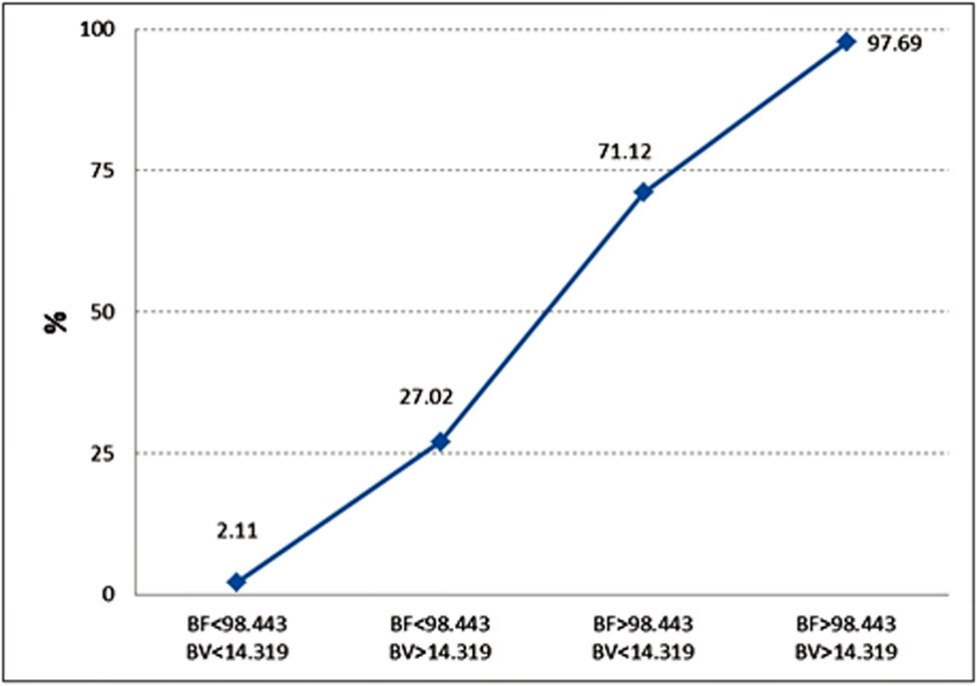
Estimated probability for the presence of PDAC.

The logistic regression model was adapted to reveal the independent diagnostic criteria of PDAC. Four parameters – BF, BV, MTT, and PS – were included in the stepwise analysis to establish the most important ones (Table 4 and Figure 5), revealing BF and BV as significant independent discriminators.

**Table 4:** Logistic regression for estimating the probability occurrence of PDAC

	*B*	S.E.	Sig.	Exp (*B*)	95% C.I. for Exp (*B*) lower	95% C.I. for Exp (*B*) upper
BF cutoff	4.737	0.908	<0.001	114.060	19.223	676.765
BV cutoff	2.842	0.959	0.003	17.148	2.618	112.324
Constant	−3.836	0.911	<0.001	0.022		

Abbreviations: BF, blood flow; BV, blood volume.

The graph shows the prognosticated probability of PDAC (%) (determined by logistic regression analysis) if the BF parameter, the BV parameter, or both of them exceed the defined cutoff value (BF < 98.443 min^−1^ 100 g^−1^, BV < 14.319 mL 100 g^−1^). If both the BF and BV values are less than the determined cutoff point, the prognosticated probability for the presence of PDAC is 2.11%; if both values exceed the determined cutoff point, the estimated probability for the presence of PDAC is 97.69%.

## 4 Discussion

Perfusion CT is a relatively new imaging modality that has been progressively used for imaging of oncological patients over the last decade [11,14,15]. Based on repetitive scanning following intravenous contrast media injection, the perfusion technique allows the measurement of functional parameters of tumor vascularity: BF, BV, MTT, and PS. An increasing number of publications report perfusion values of the pancreas [16,17,18,19].

In the present study, we showed that if BF and BV values exceed the determined cutoff point, the estimated probability for the presence of PDAC reaches 97.69%.

Therefore, estimated BF and BV parameters may serve as promising independent diagnostic criteria predicting the probability of PDAC. According to Klauss et al. [20], early detection and curative resection are of utmost importance in improving the survival rates of PDAC patients. Therefore, timely diagnosis is extremely important. MDCT is the modality of choice detecting the tumor and evaluating its resectability. However, there are PDACs that cannot be determined in conventional CT. Prokesch et al. [3] found that “up to 11% of the pancreatic carcinomas were isodense, compared to the surrounding pancreatic parenchyma.” Consequently, a reliable method that allows improving the detection rate is eligible and perfusion CT may potentially serve as such a tool [2,12,21]. Perfusion CT has not been routinely used in clinical practice yet because of the lack of consensus between radiologists [17,18,19]. Despite large heterogeneity of calculated perfusion CT parameters in the majority of studies, these results comply with the main idea that BF and BV parameters in pancreatic cancer are significantly reduced. Cutoff values are different between the studies, and this may depend on different data acquisition protocols and different software packages.

Different calculation methods (one compartment analysis [maximum slope], two-compartment analysis [Patlak], deconvolution analysis) for the post-processing of perfusion CT images demonstrate differences in the calculated perfusion values between the different mathematical models [6,13,16,21,22]. To increase the reliability of CT-perfusion, optimized perfusion CT examination protocol and standardization of post-processing are essential. To show that the different models deliver comparable results, Schneeweiß et al. [2] compared different models (maximum slope + Patlak and deconvolution analysis) in their study with PDAC patients. A moderate–good correlation between perfusion parameters in the different models was found; however, the maximum slope model underestimated BF perfusion. CT perfusion imaging develops rapidly, regardless of these significant perfusion parameter differences between the different models [2,6,12,16,17,20,21,22,23,24,25,26,27].

We calculated the median BF value of 41.74 (mL min^−1^ 100 g^−1^) and the mean BV value (SD) of 7.941 (4.315) (mL 100 g^−1^) using the deconvolution model. These results were quite different as compared with the elsewhere published results reporting BF and BV values in the tumorous tissue of 29.5 (mL min^−1^ 100 g^−1^) and 59.72 (mL 100 g^−1^), respectively, both measured with the deconvolution model [24]. Meanwhile, our results are similar to those published by Aslan et al. [28], who reported the mean values of BF and BV as 31.55 (11.54) and 2.8 (1.14), respectively.

Intriguing CT perfusion parameters in PDAC have been published to show that perfusion CT was able to evaluate tumor vascularization and even monitor response to chemo- or chemo-radiotherapy [16,21]. According to Schneeweiß et al. [2], the calculated values of the BF (mL min^−1^ 100 g^−1^) (compartment analysis) were significantly lower as compared with the deconvolution model. Klauss et al. [25] have reported quite similar BF values for pancreatic adenocarcinoma, although the calculations were performed with the Patlak analysis. BV values again were significantly higher as compared with our result (38.9 vs 7.941 mL 100 g^−1^) [20]. Yadav et al. [6] reported that BF and BV perfusion parameters were reduced in pancreatic adenocarcinoma and were found to be the most reliable in radiological diagnosis of PDAC. On the contrary, Tan et al. received markedly higher BF values (SD) 60 (15.3) mL min^−1^ 100 g^−1^ using lower temporal resolution during the first pass phase [26]. Li et al. reported that “temporal resolution between the beginning of contrast media administration and the peak enhancement (using the maximum slope model) should be considered as high as technically possible in order to avoid miscount probability” [27].

Similarly, to our data, cutoff values of 19.1 mL min^−1^ 100 g^−1^ for BF and 5 mL 100 g^−1^ for BV based on ROC analysis provided optimal sensitivity and specificity for differentiating PDAC. We detected the main perfusion parameters BF and BV as the most reliable for adenocarcinoma, with cutoff values of <98.443 min^−1^ 100 g^−1^ and 14.319 mL 100 g^−1^, respectively (using the deconvolution model). The predicted risk of probability for the presence of PDAC is approximately 2.11% if both perfusion parameters do not exceed the defined cutoff values. If these results are reproducible in other centers, it could be discussed whether biopsy may be refused in case the probability of cancer is less than 3%.

### 4.1 Study limitations

The main limitations are associated with the perfusion CT method itself. First of all, patients under examination are exposed to high radiation doses and needed for intravenous enhancement. Although the radiation exposure might be overcome, shortage of standardization remains a significant problem.

Second, the coverage of the perfusion sequence is 3 cm; thus, it was not possible to examine the entire pancreas. We could not evaluate and compare perfusion parameters of the nontumorous tissue in the same pancreas; consequently, the control group was needed. Moreover, the differentiation between tumor and chronic pancreatitis is difficult. Therefore, a control group of patients with a histologically proven diagnosis of chronic pancreatitis to evaluate the difference in CT perfusion parameters would be of great value in establishing the reliability of perfusion CT diagnosing PDAC.

Finally, this was a single-center study. Multicentric studies are further needed to show and confirm the value of our data.

## 5 Conclusion

In the present study, we showed that basic CT perfusion parameters, calculated with the deconvolution model, are reliably providing the radiological diagnosis of pancreatic adenocarcinoma, even if it is isodense or hardly depicted in conventional MDCT. The estimated BF and BV parameters are valuable as independent diagnostic criteria predicting the probability of PDAC.
